# The Outlook for the Future

**Published:** 1903-01

**Authors:** 


### THE OUTLOOK FOR THE FUTURE.

The year 1903 opens with lowering skies in the financial horizon, and while the optimists are crying prosperity there are signs of weakness in the business world, indicating that all is not as prosperous as our daily press would have us believe. In the business affairs of life these financial tides must be looked for; the ebb and flow are as constant as the waters of the ocean, and are governed by laws equally as immutable. To the philosophic mind there is nothing to regret in this, for it teaches the lesson that in the affairs of the world there must be changes, and time alone can determine whether these will result in good to individuals or communities; but whether for good or ill they are always accompanied by a valuable lesson that in part compensates for the bad and serves to moderate the satisfaction which the good derived usually brings to the fortunate recipient. Dentistry will probably be affected by the condition of the business world. It invariably does suffer, for, unfortunately, it is ranked by the large majority as one of the luxuries to be cut off whenever the indications are ominous of evil. This will always be so as long as people are not fully educated up to the standard of placing dental work, not among the luxuries, but with the necessities, those things that insure health, freedom from suffering, and prolonged life. Are dentists doing their whole duty in educating the people to a proper consideration of these important truths? A few are, but upon the whole, it is feared there is a laxity of conscience in this regard that is not only lamentable, but borders very closely on criminality. He who has had a clear knowledge of that which is beneficial to his fellows and fails to give it is simply following a line of selfishness which is the farthest removed from the true professional standard.

The life of the average dentist is, unfortunately, mainly secluded from the world. He moves in a narrow circle, or rather remains in a measure passive while the circle of patients revolves around him. This naturally leads to a contracted range of thought, and forces the conclusion that the day's work completes his duty. He has waited on so many patients and has performed his work to the best of his ability, and nothing more can be demanded of him. He retires within himself and seeks recreation in pleasures foreign to his daily toil. While all this is natural, and in some directions commendable, is it that which is required of every one who has honorably earned the right to practise his profession? There can be but one answer to this, and that is, that the man who goes thus far and no farther, simply becomes an obstruction, a barrier to professional progress.

These thoughts may seem to some the merest platitudes, and, doubtless, they are the moral repetitions that have been wearily sounded, in other lines of work, through the centuries; but while this is true the need for this lesson is as apparent to-day as it was in the past, and is more necessary in a comparatively new profession like ours, where the tendency is to drop into a selfish vein of thought and practice. Stimulation is always necessary, so that he who reads may be influenced to make an effort to rise out of the quicksands of a selfish life that threaten to engulf him.

If individuals have been negligent in this particular, what may be said of those who are supposed to mould professional opinion? From what source does the dentist receive his professional and moral pabulum? In his undergraduate days this, doubtless, is given through his alma mater. If this mother has performed her whole duty, he should be grounded in well doing, but seed do not always fall upon fruitful soil, and, with the habitual carelessness of youth, the undergraduate eventually goes out into the world forgetting the moral training in anxiety for the financial well getting along.

The work of the teacher is thus transferred to others, and this duty falls properly to the periodical dental literature. Is it carried out there? Do we find the editors of the commercial journals endeavoring to lead the professional man, young or old, into paths that will rise upward to heights never before attained? The so-called trade journal, based as it is on commercial success, deals only with the practical details that lead to more satisfactory business results to those who own and publish it, and can never be a leader in the higher phases of professional life. The origin of these journals preclude any loftier motive, and hence the editorial management in the larger number rises no higher than the source from which they derive their inspiration.

America to-day is overwhelmed with periodicals ostensibly published in the interest of dentistry. This is, probably, not true of the majority, and it is questionable whether it is true of any.

They are all advertising media, and, as such, while often very valuable, they fail to do the work demanded by the higher thought of the profession. We need a different kind of literature. Will it be furnished?

The thought has been expressed in previous articles that it is doubtful whether this will come very speedily. The influx of the commercial kind of literature has reached flood-tide. To attract subscribers it has gone down to the dollar level. It will go still farther, and the journals will be given away just so long as capital can stand the crushing loss. The moral effect of this will be disastrous to the average dental mind. The dollar journal represents a lower standard, but to that individual it is *the* standard, the level of which is represented by the value placed upon it,—just one hundred cents. It seems evident to the writer that this low valuation cannot last, but that sooner or later there will come a demand for something higher, something more ennobling.

This journal was established to meet this want. For years, under the name of the *Independent Practitioner,* and for fourteen years under that of the INTERNATIONAL DENTAL JOURNAL, it has faithfully endeavored to raise the standard of a broader professional life, a life consistent with higher culture, dignified character, and consistent practice. It proposes to continue this, but will not descend to the methods adopted by our trade contemporaries. The importance of the dollar is recognized, for nothing can move without it, or its equivalent, but the time has arrived when it must be decided whether we are a profession or a trade, and the solution will come when the dentists of this country decide upon which side they will stand,—whether for professional or commercial progress. If for the former, then will be established a new standard of self-respect; but if for the latter, then the profession of dentistry, as such, will have ceased to exist, and in its place an aggregation of men and women whose chief thought will be to increase the size of the bank account. Is this to be the end of all the labor devoted to dentistry through several centuries? Let the answer be that, come what may, the twentieth century must not close with a dental profession dependent upon a periodical literature that represents nothing but a commercial life. Will the dentist of the period work truly towards this end? May each individual reader ponder upon the importance of this query, and then make it part of his life-work to join with others in this most important effort to establish a more elevated standard of professional thought and practice.

